# Reviews and Bibliographical Notices

**Published:** 1882-02

**Authors:** 


### REVIEWS AND BIBLIOGRAPHICAL NOTICES.

HANDBOOK OF SYSTEMATIC URINARY ANALYSIS; *Chemical and Microscopical*, by Frank M. Deems, M. D. Ph. D. New York: The Industrial Publication Company, 1881. Price 25 cents.

This valuable little work of 128 pages has just been placed upon our table by the publishers. The author claims for it simply the merit of of a compilation, arranged with a view of assisting the busy practitioner, or of giving the student systematic insight into a subject which would otherwise appear difficult and irksome. The arrangement of the work is excellent, and will enable the physician in a little time to make satisfactory examinations of any urine that may come under his observation. The author's former students will welcome this little volume with pleasure.

AN INDEX OF PHYSIOLOGY, by L. Ashley Faught, D. D. S., Philadelphia. Wm. H. Hoskins, 1881. Price $1.00.

In this little volume of 122 pages the author has summarized pretty much all that is definitely known about the science of physiology. Theories and discussions of mooted points can manifestly have no place in a work of this kind, which is primarily intended for students preparing for final examination The arrangement is novel, and commends itself at a glance. As a syllabus of physiological facts it is unequalled by any work of its size now before the public. It does not pretend to supplant any text book, but is merely an *Index*, or summary of that knowledge which had been gained by the student in his previous reading, but which has temporarily gone astray. It will doubtless be extensively used by students on the day before the final interview with the Professor of Physiology.

*The Century Magazine* for January sustains its previous reputation. The frontispiece is an engraving of Louis Adolphe Thiers, the late president of the French republic, by Cole. It is one of the most exquisite specimens of the engraver's skill. The serial novels of W. D. Howells and Mrs. Frances Hodgson Burnett, are continued and grow in interest. The most notable remaining articles are "The Revival of Burano Lace," by Catherine Cornaro; "A Provincial Capital of Mexico," by Mrs. Mary Hallock Foote, and "Oriental and

Early Greek Sculpture," by Lucy M. Mitchell, all profusely illustrated. The departments of "Topics of the Time," and "The World's Work," are filled with the usual timely and instructive essays.

*Transactions of the American Dermatological Association.* Fifth year. Chicago, 1881. Pp. 56.

The American Dermatological Association was organized in Philadelphia in the Centennial year. The *brochure* here noticed, contains an account of the work performed at the fifth annual meeting, which was held at Newport, R. I., August 30th to September 1st, 1881. The address of the President, Dr. James Nevins Hyde, of Chicago, which is given in full, is upon "*Dermatology in its Relations to Periodical Literature.*" The theme is a fruitful one, and is suggestively treated by the author. The Secretary, Dr. Arthur VanHarlingen, furnishes a full abstract of the various papers read, most of which are the results of original investigation. A list of the publications and writings of the members of the association from July, 1880, to July 1881, is appended. The number of titles entered is seventy-eight.

*Reform in Medical Education, the Aim of the Academy.* The annual address delivered before the American Academy of Medicine, by Edward T. Caswell, A. M., M. D., President of the Academy, Philadelphia, 1881. Pp. 16.

The American Academy of Medicine, is an association of medical men, who meet once a year, for the purpose either of bewailing the low standard of Medical Education, or of suggesting methods for its improvement. Six annual meetings have been held, and the Academy has got no farther than to indicate its "Aim." It is not a large body, and yet it moves slowly. Why does it keep on *aiming*, why dosen't it *hit* something?

On looking over the list of officers of the Academy, we notice the names of some members of the Council who have not been in active practice of medicine for a number of years. Is that the reason no scientific work has yet been done by the Academy?

*Twelfth Annual Report of the Manhattan Eye and Ear Hospital,* New York, 1881. Pp 48.

From this report we learn that 2,819 cases of Eye disease were treated at this institution during the year ending October 15th, 1881. It is estimated that the building and site of the new hospital are worth two hundred thousand dollars, upon which there is still an indebtedness of seventy-five thousand dollars. There should not be much difficulty in raising the required amount to clear the hospital from its debt.

*Fractures of the Inferior Maxilla*, by Dr. Thos. L. Gilmer Quincy, Ill., Toledo, O., 1881. Pp. 38. Reprint from Ohio State Journal of Dental Science.

This excellent paper upon the fractures of the lower jaw, was read at the Rock Island meeting of the Illinois State Dental Society, May 10th, 1881. It presents a condensed summary of the anatomy and treatment of this accident. It is fully illustrated by cuts from drawings made by Dr. G. V. Black of Jacksonville, Ill.

*The Quarterly Epitome of American Medicine and Surgery* for December, 1881, contains the usual number of excellent selections. The regular subscription price is $2.50. We will furnish it with the INDEPENDENT PRACTITIONER, for $5.00 per year.

*The Journal of Nervous and Mental Disease*, hitherto published in Chicago under the editorship of Drs. Jewell and Bannister, has been transferred to New York. Dr. Wm. J. Morton is the present editor, and G. P. Putnam's Sons will continue to publish it.

*The Medical Bulletin*, edited by Dr. John V. Shoemaker, has been received and placed on our exchange list. It is well edited and has an air of general prosperity about it, which is very becoming.

*The Oriental Casket* is the name of a new literary magazine to be published by L. Lum Smith, Philadelphia. Emerson Bennett is the Editor.

*Apnœa Infantum*, by Hugo Erichsen, Detroit, Mich., reprint from the *Obstetric Gazette*, September, 1881. Pp. 7.

*Missouri Medical College.* Announcement of the Post-Graduate School, 1882.

